# Draft Genome Sequence of Enterococcus plantarum Strain TRW2, Isolated from Lettuce

**DOI:** 10.1128/MRA.01428-18

**Published:** 2019-02-07

**Authors:** Inhwan You, Sukjung Choi, Thomas R. Williams, Maria L. Marco, Eun Bae Kim

**Affiliations:** a Department of Animal Life Science, Kangwon National University, Chuncheon, Kangwon-do, Republic of Korea; b Laboratory of Microbial Genomics and Big Data, College of Animal Life Sciences, Kangwon National University, Chuncheon, Kangwon-do, Republic of Korea; c Department of Food Science and Technology, University of California, Davis, California, USA; d Division of Applied Animal Science, Kangwon National University, Chuncheon, Kangwon-do, Republic of Korea; Broad Institute of MIT and Harvard University

## Abstract

We report the draft genome sequence for Enterococcus plantarum strain TRW2, isolated from the phyllosphere of romaine lettuce. The draft sequence consists of 3,383,441 bp, with a G+C content of 35.8% and 3,218 protein-coding genes. None of the 22,190 known antibiotic resistance genes were detected.

## Abstract (precis)

We report the draft genome sequence for Enterococcus plantarum strain TRW2, isolated from the phyllosphere of romaine lettuce. The draft sequence consists of 3,383,441 bp, with a G+C content of 35.8% and 3,218 protein-coding genes.

## ANNOUNCEMENT

Enterococci, well-known opportunistic pathogens that are associated with numerous human infections, are naturally found in the gastrointestinal tracts of humans and animals in various environments (1). However, plant-associated *Enterococcus* strains have been investigated in a limited number of studies (2, 3). Lettuce is one of the most popular vegetables to be consumed raw. Characterization of microorganisms associated with lettuce is important for food safety, especially unknown species that are associated with plants.

Strain TRW2 was isolated from romaine lettuce grown in the Salinas Valley of California as part of the 100K Pathogen Genome Project (4), and this genome sequence was also part of the 100K Pathogen Genome Project using previously published methods (5–7).

The sonication was performed in sterilized peptone water to obtain bacteria from the lettuce. Genomic DNA was isolated from a single colony from a de Man-Rogosa-Sharpe (MRS) agar plate and inoculated into MRS broth overnight at 30°C under anaerobic conditions. DNA was extracted using a whole-genome isolation kit (Qiagen, Valencia, CA). Fragmented genomic DNA was used for the library construction with the KAPA high-throughput (HTP) library preparation kit (catalog number KK8234, Boston, MA) on the Agilent Bravo NGS workstation (Santa Clara, CA). Library quantification was performed using the KAPA library quantification kit (catalog number KK4824).

The paired-end sequencing was performed with the BGI@UCDavis sequencing center (UC Davis, CA) using the Illumina HiSeq 2000 platform with PE100 (San Diego, CA). The 100-bp paired-end reads were assembled after being quality filtered with an in-house Perl script. Briefly, 4,195,682 reads (length ≥ 70 bp) representing 262-fold coverage of the genome sequence, in which 95% of bases showed a quality score of 31 (Illumina 1.8+), were selected and assembled using Ray 2.3.1 with a k-mer size of 31 (8). The assembled genome sequence was annotated with the Rapid Annotation using Subsystem Technology server databases and the NCBI Prokaryotic Genome Annotation Pipeline (9, 10).

**FIG 1:**
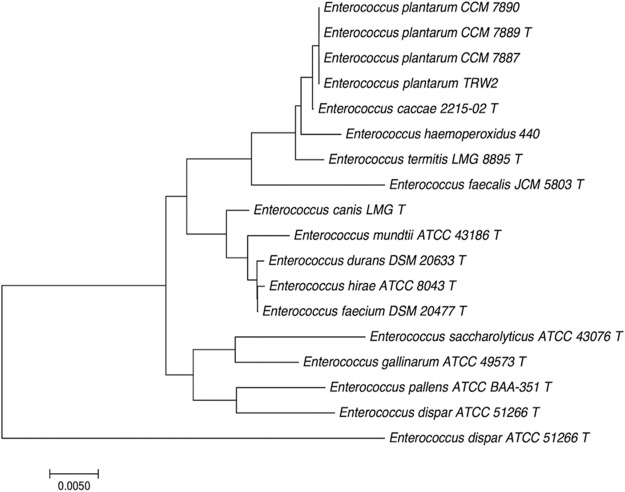
Overview of the Enterococcus plantarum TRW2 genome. 16S rRNA phylogenetic tree based on 16S rRNA gene sequences in other enterococcal species. Lactobacillus plantarum ATCC 14917 was included as an outline; T means type strain. Phylogenetic analysis was performed with the neighbor-joining method with MEGA version 7.0 (13), with a 1,000-bootstrap analysis after sequence alignment with ClustalW (14). The TRW2 strain clustered with three other E. plantarum stains.

To identify the species of strain TRW2, 16S rRNA sequences, the *sodA* gene, and the genome sequence were compared with those of other *Enterococcus* species (Fig. 1). The most similarities were found with E. plantarum (99.93% for 16S rRNA, 98.7% for the *sodA* gene, and 86.3% for the genome sequence), which indicates that the species of the strain is E. plantarum.

The properties of the E. plantarum TRW2 draft genome sequence are presented in Table 1. The draft sequence consisted of 132 contigs (≥500 bp), including 3,383,441 bp with a G+C content of 35.8% and *N*_50_ length of 89,597 bp.

**TABLE 1:** Genome sequence statistics for *E. plantarum* TRW2

Attribute	Value
Genome size (bp)	3,383,441
DNA coding (bp)	2,805,327
DNA G+C content (bp)	1,211,271
No. of DNA scaffolds	134
No. of total genes	3,409
No. of protein-coding genes	3,218
No. of RNA genes	60
No. of pseudogenes	131
No. of genes with function prediction	2,484
No. of genes assigned to clusters of orthologous groups	2,951
No. of genes with Pfam domains	2,478
No. of genes with signal peptides	3,413
No. of genes with transmembrane helices	728
No. of CRISPR repeats	2

Strain TRW2 has none of 22,190 known antibiotic resistance genes which were collected in the Comprehensive Antibiotic Resistance Database (CARD) (11), as well as none of the known enterococcal virulence factor (VF) genes analyzed by a previously reported method (12). These present results contribute to an initial description of the genomes of the E. plantarum group.

### Data availability.

The genome sequence of E. plantarum strain TRW2 has been deposited in NCBI GenBank under BioSample number SAMN07453839 and BioProject number PRJNA397329. Sequence data have been deposited in the Sequence Read Archive under the accession number SRP166833.
